# Effect of Repeated Freeze–Thaw Cycles on Influenza Virus Antibodies

**DOI:** 10.3390/vaccines9030267

**Published:** 2021-03-17

**Authors:** Alessandro Torelli, Elena Gianchecchi, Martina Monti, Pietro Piu, Irene Barneschi, Carolina Bonifazi, Rosa Coluccio, Luisa Ganfini, Luciano Michele La Magra, Silvia Marconi, Ginevra Marzucchi, Ramona Pace, Laura Palladino, Bernardo Biagi, Emanuele Montomoli

**Affiliations:** 1 VisMederi srl, 53100 Siena, Italy; Elena.Gianchecchi@vismederi.com (E.G.); Martina.Monti@vismederi.com (M.M.); Pietro.Piu@vismederi.com (P.P.); Irene.Barneschi@vismederi.com (I.B.); Carolina.Bonifazi@vismederi.com (C.B.); Rosa.Coluccio@vismederi.com (R.C.); Luisa.Ganfini@vismederi.com (L.G.); Luciano.LaMagra@vismederi.com (L.M.L.M.); Silvia.Marconi@vismederi.com (S.M.); Ginevra.Marzucchi@vismederi.com (G.M.); Ramona.Pace@vismederi.com (R.P.); laurapalladino_2013@libero.it (L.P.); bernardo.biagi@vismederi.com (B.B.); or emanuele.montomoli@vismederi.com (E.M.); 2 Department of Life Sciences, University of Siena, 53100 Siena, Italy; 3 Department of Molecular and Developmental Medicine, University of Siena, 53100 Siena, Italy

**Keywords:** Serum, stability, storage, serological test, influenza vaccine

## Abstract

*Background:* Vaccine effectiveness relies on various serological tests, whose aim is the measurement of antibody titer in serum samples collected during clinical trials before and after vaccination. Among the serological assays required by the regulatory authorities to grant influenza vaccine release there are: Hemagglutination inhibition (HAI), microneutralization (MN), and Single Radial Hemolysis (SRH). Although antibodies are regarded to be relatively stable, limited evidences on the effect of multiple freeze–thaw cycles on the stability of antibodies in frozen serum samples are available so far. In view of this, the present paper aimed to evaluate the impact of multiple freeze–thaw cycles on influenza antibody stability, performing HAI, MN and SRH assays. *Methods:* Ten serum samples were divided into 14 aliquots each, stored at −20 °C and taken through a total of 14 freeze–thaw cycles to assess influenza antibody stability. Each assay measurement was carried out following internal procedures based on World Health Organization (WHO) guidelines. *Results:* No statistically significant effect of 14 freeze–thaw cycles on antibody stability, measured through three different assays, was observed. *Conclusions:* Collectively, these data demonstrated that specific influenza antibody present in serum samples are stable up to 14 freeze–thaw cycles.

## 1. Introduction

The development cycle of a vaccine is a long and complex process from vaccine formulation until its licensing, following a multi-step approval process wherein its efficacy and safety are investigated [1]. Concerning influenza vaccine, in order to ensure matching between the viruses most likely to circulate in the upcoming season and the strain present in the vaccine, the process of selecting the viral strain to be included in the seasonal vaccine formulation constitutes a critical process. Such process is performed twice per year by the WHO. This allows to provide separate recommendations for each hemisphere on the basis of information on human influenza virus epidemiology provided by the Global Influenza Surveillance Network (GISN) [2]. Because of the frequent emergence of novel influenza variant strains, the antigenic composition of influenza vaccines needs to be reformulated almost every year to induce a sufficient protection and hence re-evaluated in terms of immunogenicity. License approval for new seasonal inactivated influenza follows the same general approach as licensure of other vaccines, aimed to evaluate vaccine safety, efficacy, and potency, necessary for marketing authorization [3].

The approval criteria for some regulatory processes in vaccine release use hemagglutinin (HA) specific antibody levels as a correlate of protection or surrogate marker for HA-based vaccine efficacy [3]. Several are the assays used for the evaluation of the immune response and they rely on the measurement of HA antibody levels in serum samples collected from vaccinees. Among the assays, HA Inhibition assay (HAI), virus neutralization assay performed as micro-neutralization (MN), and Single Radial Hemolysis (SRH) are the most used, even though not all are considered by the different international regulatory agencies mandatory for vaccine registration [4]. 

Each assay allows the identification of specific sets or subsets of antibodies. In more detail, HAI assay detects antibodies able to prevent red blood cells (RBCs) and virus HA agglutination, by blocking the receptor-binding site [5]. MN assay identifies virus specific neutralizing antibodies against HA including epitopes in HA stem region. The conventional MN test is based on the inhibition of cytopathogenic effect formation in mammalian cell culture, resulting in a laborious and slow assay. To overcome these limits, it is possible to perform the assay in combination with an ELISA to detect virus-infected cells. This allows to measure different class-specific IgM, IgA, and IgG antibodies in response to influenza vaccination/infection other than HA stalk-specific antibodies [6,7,8]. Finally, SRH is based on the passive hemolysis of RBCs, mediated by complement and induced by the antibody–antigen complex formation [6,9]. 

Each assay detects anti-HA antibodies in serum samples to evaluate the antibody response towards influenza virus in study subjects’ pre-and post-influenza vaccination. Serum samples are usually collected and stored in biobanks for long time to be processed once the sample collection is ended. To note that, since the same sample can be reanalyzed in order to confirm previous results or to perform further analysis, it is normal practice to prepare single usage aliquots before freezing. However, this practice could lead to additional issues related to the number and volume of the aliquots to be managed and stored, that might enhance the cost of the clinical study. Although for most of the vaccines licensed so far, the assessment of their ability to induce a selective immune response is based on the measurement of antigen-specific antibodies in the serum samples collected from the vaccinees, as far as we know no data on the effect of multiple freeze–thaw cycles on the stability of anti-influenza antibodies in stored serum samples are available. Only data on immunoglobulins, lipoproteins, serum chemical analytes, and other components are available, lacking data on anti-HA antibodies [10,11,12,13,14,15,16]. 

The present study investigates the effect of multiple freeze–thaw cycles on influenza antibody stability, measured by HAI, MN, and SRH, the three most common assays used for the approval and licensure of influenza vaccines by regulatory.

## 2. Materials and Methods

### 2.1. Virus Antigen

A/California/07/2009 (H1N1) seasonal influenza strain was obtained from VisMederi Research (Siena, Italy). Live virus was used for HAI and MN assays, whereas an inactivated preparation was used for SRH. The virus used was propagated in cell culture.

### 2.2. Cell Culture

Madin Darby Canine Kidney (MDCK) cells were purchased from American Type Culture Collection (ATCC) and maintained for a maximum of 25 passages in Minimum Essential Medium (MEM) containing 10% fetal bovine serum (FBS), 2 mmol/L L-glutamine, 1% non-essential amino acid solution, and 100 U/mL penicillin-streptomycin. The MDCK cell cultures were grown at 37 °C in 5% CO_2_ in a humidified atmosphere and split twice a week.

### 2.3. Serum Samples

Ten serum samples were selected from the sera collection of Vismederi (Siena, Italy) using the simple randomization method. Sera samples were anonymously collected as part of routine medical checks and research projects, stored as residual sera in compliance with Italian ethics law. The study was conducted in accordance with the Declaration of Helsinki and informed consent to use the samples for research was obtained from all subjects involved in the study. VisMederi received sera anonymized without any personal information.

The following procedure was set up to generate aliquots exposed to a different number of freeze–thaw cycles before testing. Serum of each subject was aliquoted into fourteen 1.5 mL tube aliquots (named from D1 up to D14, corresponding to the day of thawing). Briefly, on day 0 the aliquots were prepared and frozen. The samples were placed at −20 °C for 24 h before the cycles of freezing/thawing started. On day 1, all but one of the aliquots for each sample were completely thawed at room temperature (RT) for 4 h and then immediately re-frozen at −20 °C. On day two, all but two of the aliquots from each sample (the one from day one and a new one) were thawed and then re-frozen. These cycles were repeated each day until day 14. In total, 140 different samples were tested by MN, SRH and HAI assays in two independent tests performed by two different operators.

Anti-A/California/07/2009 like HA Serum (H1N1, lot 16/114) influenza sheep hyperimmune serum sample provided by National Institute for Biological Standards and Control (NIBSC, London, GB) was used as positive control. Human serum without IgA, IgM, and IgG was used as negative control (Sigma-Aldrich, St. Louis, MO).

### 2.4. HAI Assay

HAI was performed as previously described [4]. Except where specified, all the other reagents were purchased from Euroclone (Pero (MI), Italy). Briefly, all serum aliquots were treated with receptor destroying enzyme (RDE) (Denka Seiken, Tokyo, Japan) and incubated over night at 37 °C. Any remaining RDE in the sample was inactivated by heating (56 °C, 1 h), before treatment with 12.5% red blood cell (RBC)-PBS solution (1 h, 4 °C). Treated sera were centrifuged and collected. A back titration of the virus of interest (A/California/07/2009) was performed prior to start the assay and the virus solution was diluted to contain 4 HAU (hemagglutination units) in 25 µL. Treated sera were two-fold diluted from 1:1 to 1:2048 directly in the 96-multiwell plate, before adding the diluted virus. Finally, 50 μL of a 0.5% RBC solution were added to each well for the evaluation of the presence of agglutination inhibition. Results were reported as HAI titer represented by the reciprocal of the highest serum dilution in which agglutination was still completely inhibited. 

### 2.5. MN Assay

MN test measures influenza virus-specific neutralizing antibodies in serum samples using a MDCK cell matrix. The readout performed for the MN method is based on the detection of the viral NP expressed by the infected cells in the monolayer. The procedure of MN assay was previously detailed [4] and here reported with minor modifications. 

Briefly, serum samples were heat inactivated (30 min, 56 °C), before being two-fold diluted from 1:1 to 1:2048 directly in EMEM medium supplemented with 2 mmol/L L-glutamine, 100 U/mL penicillin-streptomycin and 0.5% of FBS in a 96-multiwell plate. A/California/07/2009 virus, which was previously titrated, was diluted in order to obtain 100 TCID50/50 µL in the assay. Fifty μL of the viral solution were added to each well with the exception of cell control (4 wells used as negative control) and incubated for 1 h at 37 °C + 5% CO_2_ before the addition of 100 μL of MDCK cell suspension (1.5 × 10^5^ cells/ml supplemented with 2 μg/mL TPCK) in each well. All cell culture media were purchased from Euroclone (Pero (MI), Italy), whereas all chemical reagents used for MN were from Sigma Aldrich (St. Louis, MO). The plate was incubated for 18 h at 37 °C + 5% CO_2_. The following day the plates were washed twice with PBS and then the cells were fixed for 10 min with 80% of acetone solution in PBS. Aspecific binding sites were blocked with non-fat dried milk before the addition of the primary and secondary detection antibodies. Mouse Anti-Influenza A Monoclonal Antibody MAB 8257 (clone name: A1) and Anti Influenza A Monoclonal Antibody MAB 8258 (clone name: A3) were from Merk-Millipore (Darmstadt, Germany). Goat anti-mouse IgG conjugated to horseradish peroxidase (HRP) was from Bio-Rad (Segrate (MI), Italy). The detection substrate (o-Phenylediamine dihydrochloride, OPD) for the enzyme conjugated to the secondary antibody was added, blocked with sulfuric acid and optical density (OD) was read at 490 nanometer (nm) by using an automated ELISA reader. Once calculated the infection cut off (2 × (average OD of control cell wells)), to obtain the neutralization titer the OD values were converted in positive or negative anti-influenza antibody titer according to Spearman-Kärber formula. The neutralizing antibody titer represents the reciprocal of serum dilution corresponding to that well wherein at least 50% neutralization antibody titer was obtained. 

### 2.6. SRH Assay

SRH assay was carried out following the original procedure from Schild [6] with minor modifications. Before performing the test, serum samples were heat inactivated (30 min, 56 °C). Once the mixture agarose/antigen/RBC was prepared, Guinea Pig Complement (GPC) was added and SRH plates were prepared. Prior to seeding 6 µL of undiluted serum samples and controls, holes were made in each plate by using a calibrated punch. After an overnight incubation at 4 °C, the plates were incubated at 37 °C for 90 min and the results were obtained by reading the diameters of the hemolysis areas in millimeters (mm) with a calibrated viewer. Results were reported as area of hemolysis (mm^2^), proportional to the concentration of influenza antibodies. All chemical reagents used SRH were from Sigma Aldrich (St. Louis, MO, USA). Turkey Blood at 50% in Alsever buffer and Guinea Pig Complement were purchased by Emozoo (Siena, Italy).

### 2.7. Statistical Analysis

All statistical analyses were performed using Software R version 4.0.3. In order to be included in the data analysis, all the results had to meet internal quality control criteria of the assays. More specifically, for HAI and MN assays the differences among duplicates for each sample had not to be higher than 2-fold, whereas for SRH, a bias-corrected and accelerated bootstrap interval (BCA) method was calculated to estimate the 95% confidence interval (CI95%) among the geometric mean (GM) obtained from day 1 testing (GM1 = 29.79). The limits of this interval were then normalized with respect to the geometric mean GM1. In case a sample did not met the internal quality control it was re-tested and if the internal quality control was not met even after the retest, the sample was discarded from the statistical analysis.

In addition, the criterion used to evaluate the stability was that each serum samples had to meet the internal quality control. More specifically, the highest value had not to show more than 2-fold increase difference respect the lowest one, in particular each serum sample result for each thawing cycle had to be in a 50–200% range vs. control condition (in this case day 1, for each assay). In case one of the samples, for one of the results obtained for a given thawing cycle, showed a result higher than the previously described criterion, the sample was considered stable until that cycle number, and data are included into the statistical analysis. Moreover, samples which had a negative titer (5 for HAI and MN and 3997 for SRH) for each aliquot analyzed, have been excluded by statistical analysis. Standard statistical calculations have been used as detailed below. For each sample, two measurements of the titer were collected on a daily basis for 14 days (one day for each freeze–thaw cycle), and the geometric means titer (GMT) for each sample for each day was then calculated. The GMTs obtained on Day 1 constituted the reference values to which the GMTs of subsequent days were compared. Specifically, for the normalization of the GMT vectors, the GMTs of the Day 1 were set as the base (GMT1 = 100), and the index numbers of the other days, GMT_norm_(k) where k = 2, 3, …14, were calculated as:

GMT_norm_(k) = (GMT_k_/ GMT_1_) × 100 (1)

%change = ((GMT_k /_ GMT_1_) −1) × 100. (2)

This data transformation allowed aggregation and comparison of measurements obtained from independent samples. The null hypothesis was that the freeze–thaw cycles did not affect the titer over time with respect to Day 1 results. In order to verify this hypothesis Wilcoxon signed rank tests were performed comparing the GMTnorm(k) medians to the base value of 100.

## 3. Results

The serum aliquots were tested through HAI, MN, and SRH assays to evaluate the stability of HA antibodies in serum sample following 14 freeze–thaw cycles. All data were collected and internal quality control criteria were verified before performing statistical analysis. Results of each subject (numbered from 1 to 10) reported as GMT obtained from HAI, MN and SRH assays were reported in Table 1 (column A, B, and C, respectively). The values observed from each sample aliquot did not differ by more than 2-fold (50–200%) from each other when tested by HAI and MN. All the GMs calculated across testing days (from Day 2 to Day 14) were within the reference CI95% for SRH results. These findings confirmed the results obtained through the Wilcoxon signed rank test (i.e., across Day 2-Day 14 the differences observed in the GMs of area of hemolysis compared to the GM values of Day 1 were not statistically significant). Negative titers (GMT: 3.997) were obtained from SRH assay when all the aliquots generated by 3 samples (6, 7, and 8) were tested and because of that their results were excluded by the SRH assay statistical analysis. It is worth noting that these three samples had a low titer also in HAI assay (≤40, titer characterizing non-protected subject); results were confirmed also in MN for only sample number 6 (Table 1). Finally, sample number 4 was included in the statistical analysis since its HAI titer lies in 5–10 value range (Table 1). Here we did not speculate about protection as we did not have information about vaccination status and it did not represent an objective of this study.

In order to determine differences among the antibody titers obtained from Day 1 to Day 14, for each test the GMT result obtained from Day 1 was considered as reference and it was used to normalize the results (Table 2). The differences in the normalized GMTs obtained from the analysis of each day (from Day 2 to Day 14) compared to the base value did not exceed the limits of acceptability for HAI, MN, and SRH assays (Table 1, A, B, and C, respectively). All the normalized results were reported in Table 2. The Day 1-column is the reference value with all values equal to 100. The colored–shaded values are the column-wise GMs of the index numbers. The deviations from the target value of 100 (ΔGMT) were all lower than 20.

In particular, the largest differences were observed at Day 3 (ΔGMT = −19.48) for HAI, at Day 10 (ΔGMT = −15.91) for MN and at Day 13 (ΔGMT = 3.62) for SRH. None of the ΔGMT was found significant for each assay (see the *p*-value row in Table 2). To corroborate these findings, % change was calculated and, as expected, results were comparable to the highest values obtained for ΔGMT: At Day 3 −17.23% for HAI; at Day 10 −10.54% for MN and at Day 13 4.03% for SRH.

## 4. Discussion

For the first time the effects of repeated freeze -thaw cycles on anti-HA antibody stability in human serum samples have been evaluated by using three different serological assays (HAI, MN and SRH) and expressed as antibody titer. Since equivalent results in HA titer were obtained from testing different aliquots of the same subjects exposed to up to 14 freeze–thaw cycles, we assumed that HA antibody structural integrity was preserved and not affected by repeated freeze–thaw cycles. The maintenance of serum samples under optimal storage conditions and therefore the preservation of antibodies recognizing specific infectious disease antigens, especially upon freezing and thawing cycles that could occur in case of retest or additional analysis, have a relevant importance. Frozen serum banks represent a meaningful source of scientific and clinical information not only in vaccine research but also in clinical and epidemiological studies. The group of Cao [17] reported that the freezing of proteins in aqueous solution induced a damage (denaturation) in addition to the one caused by recrystallization process during thawing [17]. In view of this, it is possible to suppose that freeze–thaw cycles of serum samples may induce a damage of antibodies affecting both the measurements and the results of research. Several studies have investigated if repeated freeze–thaw cycles could affect the stability of different serum components. Nevertheless, only a few studies indicated potential deleterious effect of multiple freeze–thaw cycles on the levels of cancer, blood, or tissue biomarkers present in serum sample [18,19,20]. Other investigations reported no alteration in blood-based biomarkers of Alzheimer’s disease [21], endocrine parameter [22], and reproductive hormones following repeated freezing and thawing [23]. Even more interestingly, as observed by Pinsky et al. [12], no statistically significant effect on measles, mumps and rubella virus antibody levels measured by enzyme-linked immunoassays (EIA) was observed upon 10 freeze–thaw cycles [12]. In addition, no significant differences in IgA, IgG and IgM antibody levels recognizing *M. pneumoniae*, *Y. enterocolitica,* and Salmonella antigens between unfrozen and frozen sera after 30 freeze–thaw cycles were found by the group of Rastawicki [24], reporting only a small variation in the level of antibodies due to the antigen used in the ELISA test. In the present study, the direct comparison of HA titer baseline value obtained from Day 1 to all the other values obtained upon repeated thaw–freeze cycle (D2-D14) did not show significant changes in HA titer levels for all the assays used. Anti-influenza antibody levels were measured using two mandatory laboratory assays (HAI and SRH) requested by regulatory agencies for the evaluation of influenza vaccine efficacy and one not compulsory test (MN). The use of three serological assays is due to the fact that they rely on different principles to detect anti-influenza antibodies in serum samples allowing the identification of different sets or subsets of antibodies. In particular, the HAI assay detects antibodies able to bind to the viral HA preventing the virus-RBC agglutination and blocking the receptor-binding site. The SRH assay recognizes not only antibodies against the surface glycoproteins but also those against the internal antigens, whereas MN can identify functional neutralizing antibodies. Hence, the present study has allowed to confirm that no differences in the level of influenza anti-HA antibodies were obtained independently by the method used and that they were stable up to 14 freeze–thaw cycles. The value of HA titer obtained from each sample tested in HAI, SRH, and MN assays met the internal quality controls. Even though differences in HA titer were in some cases observed when the same sample was analyzed by using different tests, we demonstrated a positive strong correlation among HAI, SRH, and MN assays for both A and B strains, in particular between HAI and MN assays [4]. The small number of subjects included in the present investigation as well as the limit of freeze–thaw cycles for which influenza antibody titers are not impacted can represent the limits of this study.

## 5. Conclusions

To date, no study has assessed the stability of anti-influenza antibodies in serum samples. Our results are consistent with previous findings regarding the measurement of specific immunoglobulins in serum samples exposed to repeated thaw–freeze cycles. In conclusion, our research demonstrates that anti-influenza antibodies measured in serum samples were stable up to 14 thawing and freezing cycles, evaluated through the measurement of HA titer using three different serological assays. This constitutes a meaningful finding allowing us to ensure the quality of the data regarding antibody levels obtained from multiple thaw–freeze cycle of the same aliquot, a need that can occur in routine practice. This would be relevant also for the use of frozen banked specimens whose assessment could be useful for following seroepidemiological investigations and can suggest the possibility to freeze–thaw serum samples for the execution of MN assay to obtain data to support other vaccines’ release.

## Figures and Tables

**Table 1:** Day 1 geometric means titer (GMT) of each serum sample evaluated though HA Inhibition assay (HAI), micro-neutralization (MN), and Single Radial Hemolysis (SRH) assays. For HAI and MN 5 was considered a negative value, whereas for SRH 3.997 was considered negative.

Samples Tested	(A) HAI Titer	(B) MN Titer	(C) SRH Titer
1	113	453	59.788
2	57	113	39.584
3	5	5	3.997
4	5	20	11.486
5	20	40	17.165
6	20	20	3,997
7	20	80	3.997
8	20	57	3.997
9	80	160	50.258
10	5	5	3.997

**Table 2:** HAI, MN, and SRH assay: GMT normalized (base: GMT day 1 = 100).

HAI														
	Day													
Sample	1	2	3	4	5	6	7	8	9	10	11	12	13	14
1	100.00	70.71	100.00	70.71	70.71	70.71	70.71	141.42	70.71	70.71	70.71	70.71	70.71	70.71
2	100.00	141.42	70.71	70.71	70.71	70.71	70.71	70.71	70.71	141.42	70.71	70.71	70.71	100.00
4	100.00	200.00	100.00	200.00	200.00	200.00	200.00	200.00	200.00	200.00	200.00	200.00	200.00	100.00
5	100.00	100.00	100.00	100.00	100.00	141.42	100.00	100.00	100.00	100.00	100.00	100.00	100.00	100.00
6	100.00	100.00	50.00	50.00	50.00	50.00	50.00	50.00	50.00	50.00	50.00	50.00	50.00	50.00
7	100.00	100.00	100.00	100.00	141.42	100.00	100.00	100.00	100.00	100.00	100.00	100.00	200.00	100.00
8	100.00	100.00	70.71	100.00	100.00	70.71	100.00	100.00	100.00	50.00	70.71	50.00	70.71	100.00
9	100.00	100.00	70.71	100.00	100.00	100.00	100.00	70.71	70.71	100.00	100.00	100.00	70.71	70.71
GM	100.00	109.05	80.52	91.70	95.76	91.70	91.70	95.76	87.81	91.70	87.81	84.09	91.70	84.09
ΔGM	0.00	9.05	−19.48	−8.30	−4.24	−8.30	−8.30	−4.24	−12.19	−8.30	−12.19	−15.91	−8.30	−15.91
*p*−value		0.50	0.13	0.75	>0.99	>0.99	0.75	>0.99	0.56	>0.99	0.56	0.69	0.88	0.25
%change	0.00%	14.02%	−17.23%	−1.07%	4.11%	0.44%	−1.07%	4.11%	−4.73%	1.52%	−4.73%	−7.32%	4.11%	−13.57%
**MN**														
	**Day**													
**Sample**	**1**	**2**	**3**	**4**	**5**	**6**	**7**	**8**	**9**	**10**	**11**	**12**	**13**	**14**
1	100.00	100.00	100.00	141.42	141.42	141.42	100.00	141.42	100.00	70.71	100.00	141.42	141.42	141.42
2	100.00	141.42	141.42	141.42	100.00	100.00	100.00	70.71	141.42	70.71	141.42	141.42	100.00	100.00
4	100.00	70.71	100.00	70.71	100.00	100.00	100.00	70.71	100.00	100.00	100.00	100.00	70.71	70.71
5	100.00	50.00	50.00	70.71	70.71	50.00	50.00	70.71	70.71	70.71	50.00	50.00	70.71	70.71
6	100.00	70.71	70.71	70.71	70.71	100.00	100.00	100.00	70.71	50.00	70.71	50.00	70.71	50.00
7	100.00	100.00	200.00	141.42	141.42	200.00	141.42	141.42	100.00	141.42	141.42	200.00	200.00	141.42
8	100.00	100.00	70.71	100.00	70.71	70.71	70.71	70.71	70.71	70.71	70.71	70.71	70.71	70.71
9	100.00	100.00	141.42	200.00	100.00	100.00	141.42	200.00	100.00	141.42	100.00	100.00	141.42	100.00
GM	100.00	87.81	100.00	109.05	95.76	100.00	95.76	100.00	91.70	84.09	91.70	95.76	100.00	87.81
ΔGM	0.00	−12.19	0.00	9.05	−4.24	0.00	−4.24	0.00	−8.30	−15.91	−8.30	−4.24	0.00	−12.19
*p*−value		0.63	0.63	0.17	0.63	0.88	>0.99	0.48	>0.99	0.69	>0.99	0.94	0.48	0.84
%change	0.00%	−8.39%	9.28%	17.05%	−0.63%	7.77%	0.44%	8.21%	−5.81%	−10.54%	−3.22%	6.69%	8.21%	−6.88%
**SRH**														
	**Day**													
**Sample**	**1**	**2**	**3**	**4**	**5**	**6**	**7**	**8**	**9**	**10**	**11**	**12**	**13**	**14**
1	100.00	97.15	101.14	98.29	103.47	93.24	100.00	97.71	104.04	102.31	96.59	97.71	101.72	97.72
2	100.00	101.39	95.82	92.41	104.29	118.39	91.07	91.75	94.46	89.73	89.07	100.72	95.15	88.39
4	100.00	103.93	100.03	113.33	101.35	103.98	129.32	116.24	116.34	104.00	100.03	112.09	122.31	110.91
5	100.00	87.55	101.03	97.85	89.45	87.51	87.55	96.77	84.60	84.60	110.98	89.59	97.80	99.86
9	100.00	111.58	115.58	107.65	101.27	112.22	107.65	115.58	110.92	107.64	103.16	109.60	103.16	116.89
GM	100.00	100.00	102.51	101.63	99.81	102.42	102.11	103.11	101.42	97.24	99.70	101.61	103.62	102.26
ΔGM	100.00	0.00	2.51	1.63	−0.19	2.42	2.11	3.11	1.42	−2.76	−0.30	1.61	3.62	2.26
*p*−value	100.00	>0.99	0.44	0.81	0.63	0.81	>0.99	0.81	0.81	0.81	>0.99	0.81	0.81	>0.99
%change	100.00	0.32%	2.72%	1.91%	−0.04%	3.07%	3.12%	3.61%	2.07%	−2.34%	−0.03%	1.94%	4.03%	2.76%

## Data Availability

Data is contained within the present article.
